# Time trends and future prediction of coal worker’s pneumoconiosis in opencast coal mine in China based on the APC model

**DOI:** 10.1186/s12889-018-5937-0

**Published:** 2018-08-14

**Authors:** Yuting Li, Wei Xian, Haodi Xu, Jinbin Sun, Bing Han, Hongbo Liu

**Affiliations:** 1 School of Public Health, China Medical University, Shenyang, 110122 People’s Republic of China; 2 Disease Prevention and Control Department, Dalian Sixth People’s Hospital, Dalian, China

**Keywords:** Coal workers’ pneumoconiosis, Age-period-cohort model, Incidence trend, Prediction

## Abstract

**Background:**

The opencast coal mine is a specific mine differing from the underground mine. There are differences in the way into the ore body, the organization of production, transport technology and other aspects. This study aimed to describe the prevalence of CWP among ex-dust miners in opencast coal mines and estimate the incidence trend of CWP by APC model in the future.

**Methods:**

All opencast miners who had been exposed to dust for at least 1 year in opencast mines were enrolled in this study. The database included demographic details, occupational history records with the date of dust exposure, physical examination records and pneumoconiosis diagnosis records. An age-period-cohort (APC) model has been carried out in order to explore the effects of the age, period and cohort on the prevalence of CWP among ex-dust opencast miners.

**Results:**

8191 opencast miners were enrolled in the study, including 259 miners with CWP and 7932 miners without CWP. The incidence density of CWP would have an increasing trend in opencast mines from 2005 to 2024. The number of possible CWP patients predicted in this period was approximately 492. Of them, 275 miners could have suffered from CWP in 2005–2014 and 217 miners would suffer from CWP in 2015–2024 among the ex-dust opencast miners.

**Conclusions:**

The APC model had a goodness of fit in predicting the incidence trend of CWP in opencast coal mines. By this model, we predicted that 492 opencast miners could be diagnosed as CWP from 2005 to 2024. Therefore ex-dust opencast miners cannot be ignored and they should have regular physical examinations and detection for CWP.

**Electronic supplementary material:**

The online version of this article (10.1186/s12889-018-5937-0) contains supplementary material, which is available to authorized users.

## Background

The opencast coal mine is a specific coal mine which is different from the underground mine. The coal layers of those are near the surface and underground passages are not needed. As the opencast mining is efficient and cost-effective in the exploitation of mineral resource [1], more and more opencast mining are emerging with the rapid economic and technological development [2, 3]. At present, the opencast coal mine production occupies about 12% of the total production in China [2].

The way into the ore body, the organization of production, transport technology and other aspects in opencast mining are all different from those in underground mining [4]. Before the coal layer is excavated by a coal shovel operator, the exposed stone layers which overlie coal seams are removed by blasting and carried out by drillers, stemmers, and shot-firers. So the dust exposure environment is different between opencast coal mines and underground coal mines [5]. The opencast coal mine is not a closed space and the dust may diffuse quickly and irregularly. Dust concentrations also change in the same site. Although the average dust concentrations in the opencast coal mines usually were lower than the MSHA Permissible Exposure Limit (PEL) in all job categories, most of dust concentrations from surface mine driller areas were higher than the quartz exposure limit [6]. The dust diffusion in the open working environment could affect more miners. A study showed that the surface coal mining owing to dust could lead to more health problems than underground mining [7].

Coal workers’ pneumoconiosis (CWP) is a chronic and irreversible occupational lung disease caused by inhaling coal dust or silicon dust in production environment among coal miners. At present, the prevalence of CWP is high in China and the incidence of CWP has been still severe for a long time [8, 9]. Many studies had shown that the incidence of CWP had a rising trend [10, 11]. CWP is not fully curable, but preventable [12]. Although the Coal Mine Health and Safety Act of 1969 established the current federal exposure limit for respirable dust in underground and opencast coal mines, the surveillance system of CWP does not extend to surface coal miners. A Coal Workers’ Health Surveillance Program for 16 states with active surface coal mines showed that 2.0% of 2257 miners with > 1 year of surface mining experience had CWP (including 37 who had never worked underground), and 0.5% had PMF (including 9 who had never worked underground) [13]. Overexposure to respirable silica could result in progressive massive fibrosis among current surface coal miners [5]. So it is crucial to explore the prevalence characteristics and incidence trend of CWP for making effective measures to reduce the incidence of CWP.

The prevalence and incidence of CWP in opencast mines is different from that in underground mines in view of the open working space and the widespread diffusion of dust. The traditional methods used among underground miners are not suitable to explore the incidence trend of CWP among opencast miners. There is little research on the incidence trend of CWP in opencast coal mines [1, 13]. Thus, we tried to use age-period-cohort (APC) model to analyze and predict the incidence trend of CWP in opencast coal mines. To exactly reflect the effect of duct exposure and improve the accuracy of prediction, we added the person-years of dust exposure to fit the APC model and predict incidence density of CWP. We aimed to explore the incidence trend of CWP, identify risk groups and predict the incidence population. It helps to take preventive measures for the different risk groups to reduce possible losses.

## Methods

### Study population

The subjects in this study included all miners who were engaged in Haizhou and Xinqiu opencast mines (Xinqiu opencast mine went bankrupt in 2001 and Haizhou opencast mine went bankrupt in 2005). We investigated the miners who had been exposed to dust for at least 1 year. If the miners had changed their job, their duration of dust exposure in Haizhou and/or Xinqiu opencast mine should be more than half of the total duration of dust exposed. Each enrolled miner should have detailed records of their occupational history, physical examination, and chest X-rays or diagnosis of CWP before opencast mines went bankrupt. The ex-dust miners were also included in the study if their duration of dust exposure was more than 1 year in the opencast mines.

All data in the study were elicited from Fuxin Mining Area Social Security Administration Center. Haizhou and Xinqiu opencast mines were subordinate to this center. The demographic details and occupational history records were elicited from personnel records in the Manpower Resource Section of Fuxin Mining Area Social Security Administration Center. Physical examination records and chest X-rays or diagnosis of CWP were obtained from the Department of Industry Hygiene and Occupational Disease.

### Model introduction

This study used APC model to analyze and predict the incidence trend of CWP. The APC model is based on Poisson model to confirm the effect of age, period and birth cohort on the prevalence of CWP. It can describe the incidence or mortality through adjusting for the age, period and birth cohort at the same time and predict the incidence trend in the future. The APC model has been widely used in the fields of cancer and chronic diseases [14, 15]. The basic form of model is as follow:

1 $$ 1\mathrm{n}\left[E\left({r}_{ijk}\right)\right]=1\mathrm{n}\left({\theta}_{ijk}/{N}_{ijk}\right)=\mu +{\alpha}_i+{\beta}_j+{\gamma}_k+{\varepsilon}_{ijk} $$

where *θ*_*ijk*_ is the observed number of an event for age group *i* in period *j*, *N*_*ijk*_ denotes the total number of person years, *μ* is the intercept of the regression model, *α*_*i*_ denotes the age effect (*i* = 1,2,3,…,a), *β*_*j*_ denotes the period effect (*j* = 1,2,3,…,*p*), *r*_*k*_ denotes the cohort effect. In the analysis process, we need to calculate the group *k* of corresponding cohort (Eq. 2). *ε*_*ijk*_ is the random error.

2 $$ {k}_{ij}=a-i+j $$

The maximum likelihood method is used to estimate parameters. The goodness of fit of the model determines the prediction accuracy. The value of deviance represents the closeness of the expected and actual observations, which is an important parameter in model selection [16–18]. The value of deviance/df also shows the fitness of model, which is closer to 1.

### Grouping and reference group selection

In the model, we chose diagnosis age of patients as the age factor. Age was divided into 8 groups by 5-year internals (30–44, 35–39,…, 65–69). The period was from 1960 to 2004, which was divided into 9 groups by 5-year internals (1960–1964, 1965–1969,…, 2000–2004). The cohort was calculated by period minus age. There would be some overlapping cohorts according to the algorithm of the cohort, so we took the median of the cohort to solve this problem. When the three factors are all in the model, there will be linear dependence (cohort = period - age), which can lead to the parameter unidentified [19, 20]. To overcome this problem, we adopted the commonly used solution of constraining the cohort effect to have two reference points [21] (the cohort 1913–1917 and 1968–1972). It is worth noting that two cohorts which only contain one observation at both ends of the queue were not shown in view of the interpretation of the results and error [22]. So we had 14 cohorts (1898–1902, 1903–1970,…, 1963–1967). For the APC model, the middle groups as reference groups can obtain more stable results [23]. Finally, the age group of 45–49 years old and the period group of 1975–1979 were selected as reference groups.

### Incidence density of CWP and prediction

The opencast miners would alternate their workplace owing to the occupational characteristics. The opencast miners had different duration of dust exposure in different workplaces. It is difficult for opencast miners to reflect the level of dust exposure by dust concentration. Therefore, duration of dust exposure were used to reflect the effect of dust exposure on the opencast miners. The duration of dust exposure was the accumulation of the periods of all jobs with dust exposure for each coal miner. The person-years of dust exposure were accumulated duration of dust exposure for all miners in different periods. In the APC model, the incidence density of CWP was calculated by the ratio of incidence cases to the person-years of dust exposure which could improve more accuracy in the process of description and prediction than the incidence rate of CWP. We estimated the age, period and cohort parameters in corresponding groups to accomplish the prediction of incidence density. The possible cases of CWP were predicted by the person-years of dust exposure in different age groups.

### Statistical analysis

All data were analyzed by Excel 2007, SPSS 21.0 and SAS 9.3 (Genmod procedure). A *P-*value < 0.05 was considered to be statistically significant.

## Results

### Baseline characteristics

**Fig. 1:**
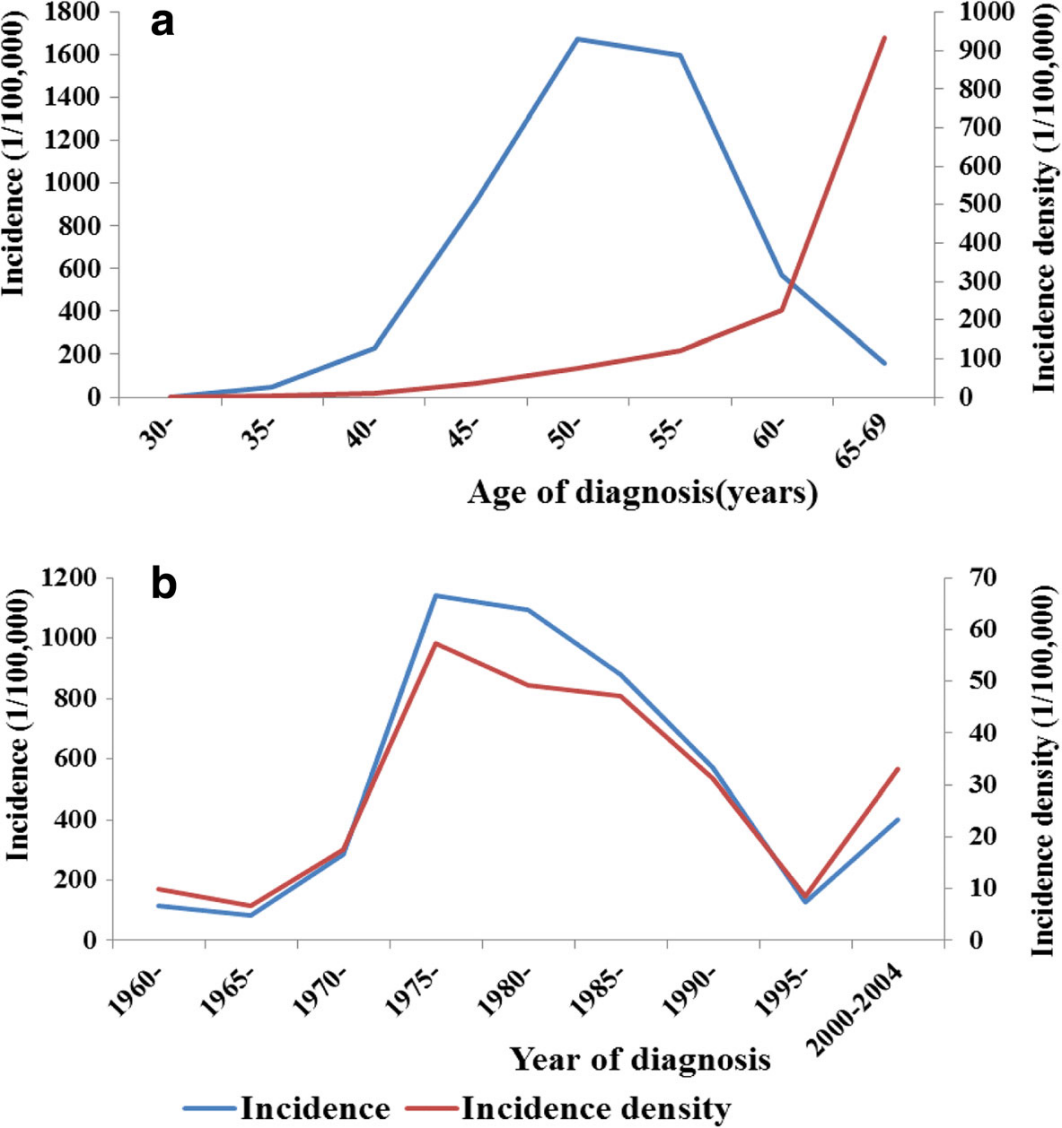
The crude incidence and incidence density of CWP in opencast coal mine (**a**. In different age groups; **b**. In different periods)

Eight thousand one hundred ninety-one opencast miners were enrolled in the study, including 259 miners with CWP and 7932 miners without CWP. Of 259 CWP patients, the average diagnosed age was 52.29 ± 5.76 years, the average exposed age was 22.75 ± 5.97 years and the average duration of dust exposure was 27.02 ± 7.37 years. For 7932 miners, the average exposed age was 21.22 ± 4.90 years and the average duration of dust exposure was 27.79 ± 8.25 years. The crude incidence of CWP increased from age group 30- to 50-, at which it went up to the highest incidence of 1671.37/100,000 (Fig. 1a). The incidence density of CWP had an increasing trend consistently from age group 30- to 65–69 (Fig. 1b). There was a similar increasing trend in the crude incidence and incidence density of CWP in different periods.

### Model selection and analysis of incidence trend

**Fig. 2:**
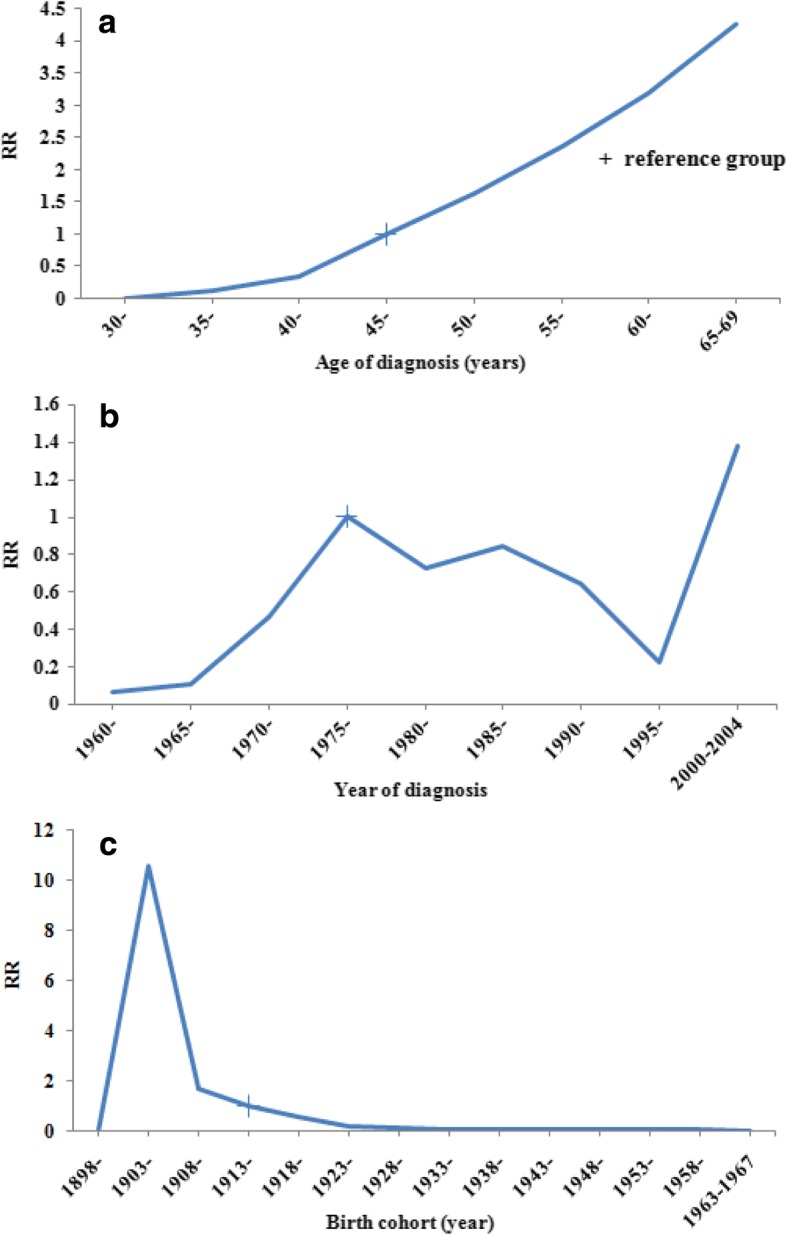
Effects of three factors in the APC model (**a**. In different age groups; **b**. In different periods; **c**. In different cohorts)

An optimal APC model was constructed in [see Additional file 1: Figure S1]. Table 1 showed that the three single-factor models could not explain the response variable (*P* < 0.05). In the three two-factor models, the AP and PC model had a good fitness (*P* > 0.05), but the fitness of AC model was not well (*P* < 0.05). In the APC model, there was a better fitting degree (*P* > 0.05). Considering the deviance among the APC, AP and PC models, we chose the APC model in this study. The effects of age, period, and cohort factors were showed in Fig. 2.

**Table 1:** Model comparison and goodness of fit for APC model analyses of CWP in opencast coal mine

Model	Deviance	df	Deviance/df	*P*
A	184.13	64	2.88	< 0.01
P	464.97	63	7.38	< 0.01
C	277.20	56	4.95	< 0.01
AP	60.93	56	1.09	0.30
AC	85.98	49	1.75	< 0.01
PC	60.34	48	1.26	0.11
APC	45.68	42	1.09	0.32

### Prediction of incidence density and possible cases

The intercept of the APC model was − 5.07. According to the different parameters in the age, period and cohort groups, we used the equation of APC model to predict the incidence density of CWP in different age groups in [see Additional file 2: Table S1]. The miners in the group 30–44 years old would have lower incidence density from 2005 to 2024. The miners in the group 55–59 years old would have the highest incidence density from 2015 to 2019. Additional file 3: Table S2 listed the sum of the person-years of dust exposure of the miners with different age group in different period [see Additional file 3]. The number of new possible CWP in 2005–2024 was listed in Table 2 and [see Additional file 4: Table S3]. If the miners in the opencast mines did have physical examination, it is possible that 492 CWP patients were diagnosed among the opencast miners who were aged from 30 to 69 years old. In the period 2005–2009, there might have been 157 new CWP patients, which mainly focus on the miners who were born in the cohort of 1938–1942. In the period 2010–2014 there might have been 118 new CWP patients which mainly focus on the miners who were born in the cohort of 1943–1947.We predicted that 103 opencast miners could be diagnosed as CWP in 2020–2024.

**Table 2:** Possible cases of CWP and incidence density in opencast miners

Predicted period(year)	Cases	Person –years	Incidence density(1/100,000)
2005-	156.54	155,517.46	100.66
2010-	118.39	118,014.91	100.32
2015-	113.11	100,914.24	112.09
2020–2024	103.33	77,796.49	132.82
Total	491.37	452,243.10	108.65

## Discussion

CWP is a serious occupational disease in China [24]. A steady decrease in the number of coal miners exposed to dust in developed countries has played a crucial role. But in developing countries, especially China, more and more miners are exposed to high concentrations of dust lacking effective surveillance and are at a high risk for CWP [25]. Although reducing production or continuing bankruptcies resulted in a reduction of miners, and many effective measurements have been used to reduce dust in workplace, there’s not a significant decrease of CWP incidence in a short time because of delayed onset of CWP. The miners who worked in a lower dust concentration or left dust environment still had a risk for CWP owing to the irreversibility of pneumoconiosis [8, 25]. At present, there are not some corresponding effective surveillances to regularly screen and manage these ex-dust exposure miners. Due to economic burden, the miners rarely went hospital for regular screening and they cannot be diagnosed as CWP in time. What’s even more frightening is that the dust in the lung cannot be removed and the incidence of CWP cannot be prohibited. So it is important to identify the risk for CWP in the opencast miners and take targeted strategies to decrease and control the incidence of CWP.

Different from the traditional methods in the study of underground coal mines, we tried to use APC model to analyze and predict the incidence trend of CWP. The APC model could reduce the limitations in the calculation of age-specific incidence rate and age-adjusted incidence rate which were usually used in traditional methods [26, 27]. It can describe the incidence trend of CWP in different ages, periods and cohorts and compare the relative risk among different subgroups. Many researchers had used the APC model to predict the future trend [28, 29]. Because the incidence of CWP was affected by cumulative level of dust exposure, we used the person-years of dust exposure to calculate the incidence density of CWP. The study helps us more accurately find the risk groups and provide a theoretical basis for the prevention and control of CWP.

In our study, the risk for CWP in opencast miners increased from 1960 to 1979, decreased gradually from 1980 to 1999, and then increased again after 2000. In the early 1960s, the opencast coal mines were mined by manual operations. The system of dust prevention was incomplete, and the awareness of self-protection was poor among opencast miners. As China’s pneumoconiosis prevention and control regulations enacted in the 1980s [30], the prevention of occupational diseases were paid attention to in many mines. The personal protection equipment and awareness of self-protection were improved, and the manual operation in mining was replaced by the machine operation. The incidence of CWP was effectively controlled and had a declining trend after taking actions. But after 2000, the two opencast coal mines gradually went bankrupt, which may lead to the increasing risk for CWP because of lack of effective management. Through the APC model, we predicted that there would be 275 new patients with CWP from 2005 to 2015. So the prevention and screening of CWP should not be ignored among the miners [31]. The awareness of personal protection, equipment improvement and strict management all played positive roles in controlling the trend of incidence of CWP.

As you know, the diagnoses of occupational diseases and the treatment and recoveries of patients should be supported by their employment company or the injury insurance according to the Law of Occupational Disease. But the miners’ health was not well monitored after the company bankruptcy. They pay the fee of screening diseases by themselves, so they seldom regularly have health examination owing to economic burden for numerous families. Finally, more miners lost chances of early detection and early treatment, resulting in greater economic losses [32]. It was reported that the direct economic losses of pneumoconiosis in China added up to 8 billion RMB each year, and the indirect economic losses was reach up to 20 billion RMB [8]. The economic or social loss would rise if effective measures are not taken. Health records of the miners should be established even if the company gone bankrupt. They should have a regular health examination and follow-up. It is useful for early diagnosis and early treatment among the miners in order to reduce the social and family burden.

There are some limitations in our study. The Haizhou and Xinqiu opencast mines are located in northeast China. Although they are typical state-run colliery in China, only two mines were studied, and may be less representative. But, it is feasible that the analysis model in this study is applied to identify the risk of CWP in opencast mines after appropriately adjusting. Additionally, dust concentration and cumulative dose exposure are the most important factors to influence the occurrence of CWP [33]. Because of the administration system and the characteristics of the working process in opencast mines, it is difficult to collect accurate and detail data of the dust measurements. Thus we considered the duration of dust exposure [34] and person-years of dust exposure to reflect dust exposure level in order to better fit the APC model and predict the incidence of CWP reasonably. We had predicted the key groups with high risk for CWP, and further health screening should be conducted. We can identify the interval of physical examination or health screening according to basic characteristics of opencast miners combining with the risk for CWP predicted by APC model.

## Conclusions

The APC model had a goodness of fit in accurately predicting the incidence trend of CWP and reflecting the potential risk for CWP in opencast coal mines. This model can compare the incidence risk for CWP in subgroups of age, period and cohort. In our study, the risk for CWP in opencast miners increased from 1960 to 1979, decreased gradually from 1980 to 1999, and then increased again after 2000. We predicted that 492 opencast miners could be diagnosed as CWP from 2005 to 2024. If the opencast miners did have physical examination, there might have been 157 new CWP patients. Therefore ex-dust opencast miners cannot be ignored and should have regular physical examinations and detection for CWP.

## Additional files

Additional file 1: **Figure S1.** Diagnostic figure of the goodness of fit in APC model. (DOC 32 kb)

Additional file 2: **Table S1.** Incidence density of CWP in different age groups (1/100,000). (DOC 35 kb)

Additional file 3: **Table S2.** Person-years of dust exposure in different age groups. (DOC 37 kb)

Additional file 4: **Table S3.** Possible cases of CWP in different birth cohorts. (DOC 39 kb)

Additional file 5: The raw data related to our study. (XLSX 1154 kb)

## Abbreviations

APC: Age-period-cohort model
CWP: Coal workers’ pneumoconiosis
MSHA: Mine Safety and Health Administration
PEL: Permissible Exposure Limit
RR: Relative risk
