# Supplementary figures and images for: Time trends and future prediction of coal worker’s pneumoconiosis in opencast coal mine in China based on the APC model
Source: BMC Public Health. 2018 Aug 14;18:1010. doi: 10.1186/s12889-018-5937-0 (PMC6092848; doi:10.1186/s12889-018-5937-0)

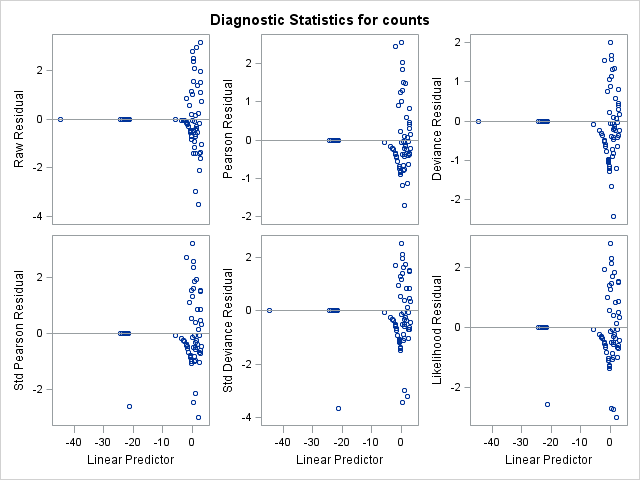
 Figure S1 Diagnostic figure of the goodness of fit in APC model

Supplement: Supplementary file 1 — Figure S1. Diagnostic figure of the goodness of fit in APC model. (DOC 32 kb) [file 12889_2018_5937_MOESM1_ESM.doc]
