# Supplementary material for: Time trends and future prediction of coal worker’s pneumoconiosis in opencast coal mine in China based on the APC model
Source: BMC Public Health. 2018 Aug 14;18:1010. doi: 10.1186/s12889-018-5937-0 (PMC6092848; doi:10.1186/s12889-018-5937-0)
Supplement: Supplementary file 2 — Table S1. Incidence density of CWP in different age groups (1/100,000). (DOC 35 kb) [file 12889_2018_5937_MOESM2_ESM.doc]

Table S1 Incidence density of CWP in different age groups (1/100,000)

| Age of diagnosis (years) | Predicted period(year) | | | |
| --- | --- | --- | --- | --- |
|  | 2005- | 2010- | 2015- | 2020-2024 |
| 30- | 0 | 0 | 0 | 0 |
| 35- | 0 | 0 | 0 | 0 |
| 40- | 0 | 0 | 0 | 0 |
| 45- | 31.59 | 0 | 0 | 0 |
| 50- | 40.16 | 56.99 | 0 | 0 |
| 55- | 88.49 | 64.26 | 91.19 | 0 |
| 60- | 178.20 | 132.02 | 95.86 | 134.68 |
| 65-69 | 226.54 | 263.20 | 194.99 | 140.18 |
