# Supplementary material for: Time trends and future prediction of coal worker’s pneumoconiosis in opencast coal mine in China based on the APC model
Source: BMC Public Health. 2018 Aug 14;18:1010. doi: 10.1186/s12889-018-5937-0 (PMC6092848; doi:10.1186/s12889-018-5937-0)
Supplement: Supplementary file 3 — Table S2. Person-years of dust exposure in different age groups. (DOC 37 kb) [file 12889_2018_5937_MOESM3_ESM.doc]

Table S2 Dust exposed person-years in different age groups

| Age of diagnosis (years) | Predicted period(year) | | | |
| --- | --- | --- | --- | --- |
|  | 2005- | 2010- | 2015- | 2020-2024 |
| 30- | 0 | 0 | 0 | 0 |
| 35- | 236.32 | 0 | 0 | 0 |
| 40- | 4859.77 | 236.32 | 0 | 0 |
| 45- | 21015.83 | 4698.09 | 236.32 | 0 |
| 50- | 54749.36 | 20422.54 | 4576.34 | 236.32 |
| 55- | 23373.77 | 54162.05 | 20076.93 | 4413.92 |
| 60- | 18497.74 | 22394.86 | 53909.57 | 19621.50 |
| 65-69 | 32784.67 | 16101.05 | 22115.08 | 53524.75 |
| Total | 155517.46 | 118014.91 | 100914.24 | 77796.49 |
