# Supplementary material for: Time trends and future prediction of coal worker’s pneumoconiosis in opencast coal mine in China based on the APC model
Source: BMC Public Health. 2018 Aug 14;18:1010. doi: 10.1186/s12889-018-5937-0 (PMC6092848; doi:10.1186/s12889-018-5937-0)
Supplement: Supplementary file 4 — Table S3. Possible cases of CWP in different birth cohorts. (DOC 39 kb) [file 12889_2018_5937_MOESM4_ESM.doc]

Table S3 Possible cases of CWP in different birth cohorts

| Birth cohort  (year) | Predicted period(year) | | | |
| --- | --- | --- | --- | --- |
|  | 2005- | 2010- | 2015- | 2020-2024 |
| 1938- | 74.27 | - | - | - |
| 1943- | 32.96 | 42.38 | - | - |
| 1948- | 20.68 | 29.57 | 43.12 | - |
| 1953- | 21.99 | 34.80 | 51.68 | 75.03 |
| 1958- | 6.64 | 11.64 | 18.31 | 28.30 |
| 1963- | 0 | 0 | 0 | 0 |
| 1968- | 0 | 0 | 0 | 0 |
| 1973- | 0 | 0 | 0 | 0 |
| 1978- | - | 0 | 0 | 0 |
| 1983- | - | - | 0 | 0 |
| 1988-1992 | - | - | - | 0 |
| Total | 156.54 | 118.39 | 113.11 | 103.33 |
